# Strings on a Violin: Location Dependence of Frequency Tuning in Active Dendrites

**DOI:** 10.3389/fncel.2017.00072

**Published:** 2017-03-13

**Authors:** Anindita Das, Rahul K. Rathour, Rishikesh Narayanan

**Affiliations:** ^1^Cellular Neurophysiology Laboratory, Molecular Biophysics Unit, Indian Institute of ScienceBangalore, India; ^2^Center for Learning and Memory, The University of Texas at AustinAustin, TX, USA

**Keywords:** active dendrites, degeneracy, impedance, intrinsic plasticity, ion channels, oscillations, resonance, spike-triggered average

## Abstract

Strings on a violin are tuned to generate distinct sound frequencies in a manner that is firmly dependent on finger location along the fingerboard. Sound frequencies emerging from different violins could be very different based on their architecture, the nature of strings and their tuning. Analogously, active neuronal dendrites, dendrites endowed with active channel conductances, are tuned to distinct input frequencies in a manner that is dependent on the dendritic location of the synaptic inputs. Further, disparate channel expression profiles and differences in morphological characteristics could result in dendrites on different neurons of the same subtype tuned to distinct frequency ranges. Alternately, similar location-dependence along dendritic structures could be achieved through disparate combinations of channel profiles and morphological characteristics, leading to degeneracy in active dendritic spectral tuning. Akin to strings on a violin being tuned to different frequencies than those on a viola or a cello, different neuronal subtypes exhibit distinct channel profiles and disparate morphological characteristics endowing each neuronal subtype with unique location-dependent frequency selectivity. Finally, similar to the tunability of musical instruments to elicit distinct location-dependent sounds, neuronal frequency selectivity and its location-dependence are tunable through activity-dependent plasticity of ion channels and morphology. In this morceau, we explore the origins of neuronal frequency selectivity, and survey the literature on the mechanisms behind the emergence of location-dependence in distinct forms of frequency tuning. As a coda to this composition, we present some future directions for this exciting convergence of biophysical mechanisms that endow a neuron with frequency multiplexing capabilities.

## Introduction

The advent of patch clamp electrophysiology allowed direct electrical access to neuronal sub-cellular compartments and paved the way for giant strides in the field of single neuron physiology with a focus on the hitherto unfound active dendritic properties (Stuart et al., 1993). Since then the portrait of a single neuron has undergone major transformations with multiple lines of research providing testimony for the role of dendrites, dendritic ion channel expression and their plasticity in single neuron computations (Johnston et al., 1996; Migliore and Shepherd, 2002; Magee and Johnston, 2005; Johnston and Narayanan, 2008; Sjöström et al., 2008; Narayanan and Johnston, 2012; Stuart and Spruston, 2015). The focus of this review article is on a specific aspect of dendritic physiology, one that critically relies on active gradients within a dendritic structure and one that endows neurons with location-dependent input processing. The demonstration of location-dependent frequency selectivity mediated by active dendritic conductances (Narayanan and Johnston, 2007, 2008; Hu et al., 2009; Kalmbach et al., 2013; Das and Narayanan, 2014) ushered in the possibility of a novel role for single neurons to detect and synchronize their activity to a frequency of an ongoing network oscillation or perform selective routing of synaptic inputs based on their spectral content (Hong et al., 2007; Buzsáki, 2010; Ratté et al., 2013; Das and Narayanan, 2015). Juxtaposed with the literature on inhomogeneous distribution of dendritic conductances, spatiotemporal interactions between them, plasticity of intrinsic neuronal properties, activity- and state-dependent modulation of expression profiles and degeneracy in neuronal physiology, we can posit a complex yet nuanced role for plastic active dendrites in spectral tuning of single neurons. The aim of this narrative is to discuss the various forms and mechanisms of spectral tuning present in neurons with the focus primarily on the role of dendrites. We highlight the several endeavors undertaken to dissect various forms of neuronal spectral tuning, deliberate over their implications for dendritic physiology and eventually propose tenable future directions for steering research in this field.

## Historical Overview of Neuronal Spectral Tuning

In a series of seminal articles, Cole and colleagues explored spectral properties of various biological substrates employing theoretical and experimental techniques. Using the squid giant axon preparation and employing multiple theoretical and analytical tools, Cole and colleagues provided the first evidence for the presence of *inductive reactance* alongside the previously known resistive element and capacitive reactance in the biological membrane (Cole, 1941; Cole and Baker, 1941a,b; Cole and Curtis, 1941). Theoretically, the presence of inductive reactance in the neuronal membrane could greatly alter the spectrotemporal relationship between current and voltage depending upon their balance with capacitive reactances (Skilling, 1965). This discovery opened up an avenue of possibilities for exploring the spectrotemporal properties of neuronal responses with novel implications for neuronal spectral selectivity and neural circuits.

Cole and others hypothesized that the presence of time-variant resistance in neuronal membrane could constitute a *phenomenological* inductive reactance. Later, experimental and modeling studies demonstrated the presence of voltage-dependent conductances that could serve the purpose, and were termed as “anomalous” or “phenomenological” inductances (Cole, 1949; Mauro, 1961; Sabah and Leibovic, 1969; Mauro et al., 1970). As research in this field progressed with important advances in experimental techniques that allowed us to directly access and characterize neuronal membrane properties, the role of various voltage-gated ion channels (VGICs) in mediating/modulating spectral tuning properties came to light (Hutcheon and Yarom, 2000).

## Different Forms of Spectral Tuning in Neurons

**Figure 1:**
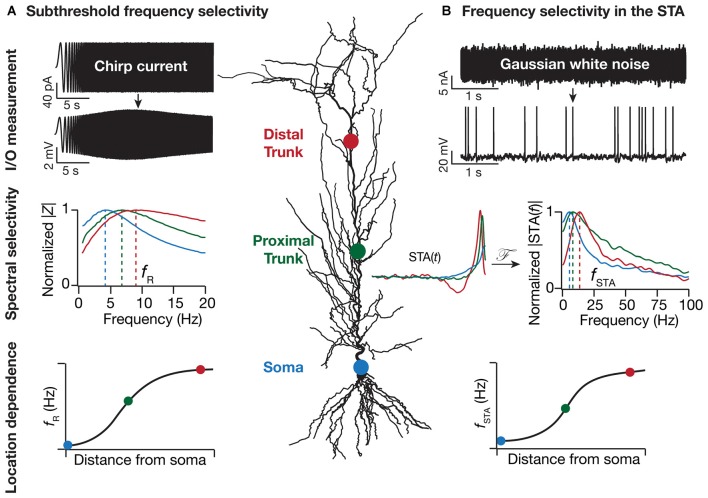
**Location dependence of two distinct forms of dendritic frequency selectivity. (A)** Resonance in local impedance amplitude (|*Z*|) profile is measured by recording local voltage responses to a chirp current injection. This resonance frequency (*f*_R_), measured at subthreshold voltages, is location dependent and increases with distance from the soma (Narayanan and Johnston, 2007). **(B)** Spectral tuning in the spike-triggered average (STA) is measured by recording somatic voltage responses to Gaussian white noise current injection. The amplitude of the STA’s Fourier transform (|STA(*f*)|), with the STA computed as the average current stimulus that elicits somatic spikes, exhibits frequency selectivity. This STA characteristic frequency (*f*_STA_) increases with distance from the soma (Das and Narayanan, 2014, 2015). With reference to both *f*_R_ and *f*_STA_, the sharpness of tuning quantified as selectivity strength, measured as the maximal response amplitude divided by the response amplitude at 0.5 Hz, increases with dendritic distance from the cell body. The normalized |*Z*| profiles were derived from electrophysiological experiments in Narayanan and Johnston (2007) whereas the |STA(*f*)| profiles were generated from the computational model in Das and Narayanan (2015). The location dependent profiles of *f*_R_ and *f*_STA_ are cartoon versions to illustrate the increase in these measurements with distance from the cell body, with data from Narayanan and Johnston (2007) and Das and Narayanan (2014, 2015).

Neurons in the central nervous system are endowed with myriad VGICs, which by virtue of their complex spatiotemporal interactions bestow neurons with subthreshold and suprathreshold spectral tuning. One of the best-studied forms of spectral tuning is *membrane potential* *resonance* characterized by a peak in the neuron’s impedance amplitude profile (Figure 1) computed over a range of input frequencies (Gimbarzevsky et al., 1984; Hutcheon and Yarom, 2000). Apart from invertebrate giant axons from where initial evidence came, direct evidence for the presence of electrical resonance also came from experiments on numerous neuronal subtypes of the central and peripheral nervous system (Crawford and Fettiplace, 1981; Puil et al., 1986; Hutcheon and Yarom, 2000; Pike et al., 2000). Subthreshold resonance in local responses and in dendrite-to-soma transfer endows neurons with the ability to discriminate inputs based upon their frequency content. While the impedance *amplitude* profile defines the voltage-current relationship in terms of maximal subthreshold response, the impedance *phase* profile quantifies the temporal dynamics between voltage and current. It has been shown that presence of inductive reactance in neuronal membrane causes the voltage response to lead the injected oscillatory current. The balance between capacitive and inductive reactances determines both the optimal lead frequency and the frequency bandwidth over which the voltage leads the current, providing a potential mechanism by which the subthreshold membrane dynamics of the neuron may maintain a phase relationship with an ongoing oscillation (Mauro, 1961; Cole, 1968; Sabah and Leibovic, 1969; Mauro et al., 1970; Hu et al., 2002, 2009; Ulrich, 2002; Cook et al., 2007; Narayanan and Johnston, 2008; Vaidya and Johnston, 2013).

While subthreshold spectral selectivity arms the neurons and their dendrites with the ability to selectively transmit information based on frequency and phase content of afferent inputs, a critical question is: does this information translate to an output that is relayed to the downstream neurons in the network? This question touches upon the conjoined problems of neural *dynamics* and *coding*, resulting in the definition of suprathreshold spectral selectivity as the spectral content of incoming signals onto the dendrites that results in effective generation and propagation of a somatic action potential. There are multiple neuronal physiological proxies for suprathreshold selectivity including firing rate resonance, spike triggered average (STA), synchrony detection and synchrony transfer (Bryant and Segundo, 1976; Haag and Borst, 1996; Joris et al., 1998; Brunel et al., 2003; Richardson et al., 2003; Hong et al., 2007; Famulare and Fairhall, 2010; Kispersky et al., 2012; Ratté et al., 2013; Das and Narayanan, 2014). Each of these aim to quantify the spectral signals that single neurons glean from their afferent inputs leading to a suprathreshold response in terms of somatic firing. The ability of neurons to detect high frequency or temporally proximal inputs is pivotal to multiple neuronal functions such as frequency multiplexing, spike phase coherence and coincidence detection (Softky, 1994; Joris et al., 1998; Colgin et al., 2009; Buzsáki, 2010; Lisman and Jensen, 2013; Das and Narayanan, 2015). While impedance amplitude analysis provides a robust quantitative metric to understand subthreshold spectral tuning in neurons, a single physiological measure to quantify suprathreshold spectral selectivity is lacking. In a primary sensory area such as the auditory cortex, neurons have been characterized according to the best or characteristic sound frequency that they fire to. For multimodal areas such as the thalamus and the hippocampus, this categorization is not straightforward as the inputs themselves are a complex pattern of excitatory and inhibitory afferents from multiple anatomical locations. To this end, the STA provides a useful tool to quantify neuronal suprathreshold spectral selectivity from the input features that result in a spike (Figure 1), and to assess the relationship between sub- and supra-threshold selectivities (Das and Narayanan, 2014, 2015).

## Biophysical Basis of Diverse Spectral Tuning Mechanisms

A number of biophysical mechanisms have been implicated in conferring robust subthreshold resonance upon neurons. As is evident from the physics of electric circuits, interplay between inductive and capacitive reactances dictates resonant behavior, where capacitive reactance along with resistive component forms a low pass filter and inductive reactance along with resistive element forms a high pass filter. A combination of the two reactances with the resistance forms a band pass filter leading to resonance. In neurons, the membrane contributes towards the capacitive reactance, distinct ion channels (prominently the leak channels) contribute to the resistance, while various *resonating* conductances, including hyperpolarization-activated cyclic-nucleotide-gated (HCN), *M*-type potassium and *T*-type calcium channels, mediate an inductive reactance. A resonating conductance satisfies two biophysical constraints: (i) the conductance opposes changes in membrane potential; and (ii) the (in)activation time constant of the conductance is slower than the membrane time constant (Hutcheon and Yarom, 2000).

With reference to suprathreshold spectral selectivity, interactions between a fast inward current and a slow outward current have been shown to determine the specific input features that could alter spike initiation dynamics. Moreover, VGICs that regulate spike threshold, repolarization kinetics, spike afterhypolarization and spike frequency adaptation alter spiking dynamics and suprathreshold frequency selectivity. Various voltage- and calcium-activated potassium channels, along with synaptic components that alter neuronal membrane excitability through network activity, have been implicated in such regulation of suprathreshold tuning. Additionally, resonating conductances, by altering the subthreshold dynamics of the neuron can translate subthreshold selectivity to the suprathreshold regime. Thus, a consortium of intrinsic and network mechanims could convert subthreshold spectral selectivity to the suprathreshold regime or evoke emergent suprathreshold selectivity dissociated from subthreshold resonance (Reyes et al., 1994; Softky, 1994; Haag and Borst, 1996; Pape et al., 1998; Hu et al., 2002; Brunel et al., 2003; Richardson et al., 2003; Badel et al., 2008; Kispersky et al., 2012; Ratté et al., 2013; Stark et al., 2013; Das and Narayanan, 2014, 2015).

## Location-, Morphology- and Activity-Dependence of Dendritic Spectral Tuning

The presence of VGICs in dendrites bestows neurons with enormous computational capabilities related to input, integration and output modules of information processing. As the distribution of various VGICs along the somato-dendritic axis has been shown to be non-uniform (Johnston et al., 1996; Migliore and Shepherd, 2002; Johnston and Narayanan, 2008; Narayanan and Johnston, 2012), it is not surprising that individual locations along this axis have distinct physiological properties and computational abilities. Given the non-uniform distribution of various VGICs and their coupling to spectral tuning properties, it stands to reason that spectral tuning itself depends heavily on the location along the somato-dendritic axis. Indeed, it has been demonstrated that HCN conductance dependent sub-threshold spectral tuning changes with dendritic location to give rise to a functional map (Figure 1). Specifically, it has been electrophysiologically and computationally demonstrated that an increase in HCN conductance along the somato-apical axis leads to an increase in the resonance frequency and inductive phase in pyramidal neurons, followed by demonstrations related to gradients in other resonating conductances (Narayanan and Johnston, 2007, 2008; Hu et al., 2009; Marcelin et al., 2009; Kalmbach et al., 2013). Spatiotemporal interactions with other VGICs expressed in the dendrites and activity-dependent plasticity in their expression profiles allows for the emergence of location-dependent and dynamically tunable subthreshold resonance along the neuronal topograph (Magee and Johnston, 2005; Narayanan and Johnston, 2007, 2008; Sjöström et al., 2008; Hu et al., 2009; Rathour and Narayanan, 2012a,b). Inhomogeneous distribution of various subthreshold VGICs, together with their interactions with spike-generating conductances along the somato-dendritic axis, can also result in distinct suprathreshold spectral tuning profiles (Figure 1; Das and Narayanan, 2014, 2015).

An additional factor that modulates neuronal spectral selectivity is dendritic arborization. While literature in this regard is exiguous, there is evidence that active dendritic mechanisms coupled with the structure and plasticity of the dendritic arbor (Softky, 1994; Mainen and Sejnowski, 1996; Agmon-Snir et al., 1998; Dhupia et al., 2015; Ostojic et al., 2015) can alter both subthreshold and suprathreshold spectral tuning. These distinct mechanisms endow single neurons with several tools to alter their spectral tuning, locally or globally, in response to varying physiological and pathological conditions (Brager and Johnston, 2007, 2014; Narayanan and Johnston, 2007, 2008; Shin et al., 2008; Marcelin et al., 2009; Narayanan et al., 2010; Brager et al., 2012; Zhang et al., 2014).

## Degeneracy in Active Dendritic Spectral Tuning

**Figure 2:**
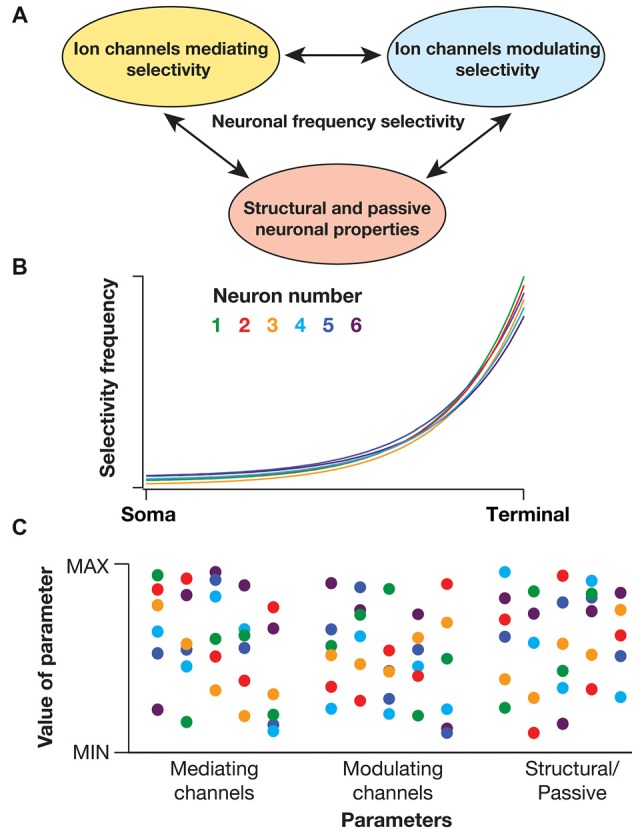
**Degeneracy in active dendritic spectral tuning. (A)** Spectral tuning in active dendritic structures are regulated by several neuronal properties. There are specific biophysical requirements on the channels that could *mediate* frequency selectivity (see “Biophysical Basis of Diverse Spectral Tuning Mechanisms” Section). However, there are strong lines of evidence that channels that do not satisfy these constraints could *modulate* frequency selectivity (Narayanan and Johnston, 2007; Zemankovics et al., 2010; Rathour and Narayanan, 2012a, 2014; Rathour et al., 2016). For instance, the inductive component and frequency selectivity that is *mediated* by hyperpolarization-activated cyclic-nucleotide-gated (HCN) or *T*-type calcium channels is significantly modulated by *A*-type potassium channel, a channel that does not satisfy the requirements for a resonating conductance (Rathour and Narayanan, 2012a, 2014; Rathour et al., 2016). In addition, morphological properties of the dendritic arbor could alter resonance and frequency selectivity (Dhupia et al., 2015; Ostojic et al., 2015). Therefore, the specific quantitative aspects of spectral selectivity emerge through synergistic interactions between channels that mediate frequency selectivity (*e.g*., HCN, *T*-type calcium), channels that modulate frequency selectivity (*e.g., A*-type potassium, leak) and morphological parameters. **(B)** Cartoon of somatodendritic spectral tuning profiles of six different neurons (each neuron depicted with a specific color code) showing similar frequency selectivity across different locations. **(C)** Despite functional similarity, parameters underlying the six different neurons depicted in **(B)** show significant variability (same color codes in **B**). These parameters span properties of channels (*e.g*., density, half-maximal activation voltage, time constants) that mediate or modulate frequency selectivity and morphological characteristics (*e.g*., length, diameter) that alter the specific quantitative aspects of spectral selectivity. As a consequence of synergistic interactions between these parameters towards yielding specific frequency selectivity, similar somatodendritic spectral tuning could be achieved through disparate parametric combinations. This implies the expression of degeneracy in active dendritic spectral tuning (Rathour and Narayanan, 2014; Rathour et al., 2016).

There are several lines of evidence that demonstrate the critical dependence of sub- and supra-threshold frequency selectivity on disparate physiological mechanisms. These mechanisms could broadly be classified into three categories (Figure 2A): (i) channels/mechanisms endowed with specific properties that allow them to *mediate* spectral selectivity; (ii) channels/mechanisms that are incapable of mediating selectivity, but that can *modulate* selectivity; and (iii) passive neuronal properties and dendritic arborization that *modulate* selectivity. How do neurons maintain robust location-dependent spectral selectivity in the face of regulation by several such parameters, with each of them exhibiting significant variability across neurons (even of the same subtype)?

Degeneracy, defined as the ability of a system to elicit similar functional outputs through disparate combinations of constituent components, proffers an ideal construct for achieving such robustness (Edelman and Gally, 2001). Such degeneracy has been observed in several neuronal systems and at various scales for bringing about robustness in distinct combinations of physiological properties (Foster et al., 1993; Edelman and Gally, 2001; Prinz et al., 2004; Marder and Goaillard, 2006; Marder, 2011; Rathour and Narayanan, 2012a, 2014; Anirudhan and Narayanan, 2015). With reference to sub- and supra-threshold frequency selectivity in neurons, several studies have demonstrated multi-channel regulation of somatodendritic spectral selectivity. Specifically, studies pertaining to sub-threshold resonance and phase dynamics have shown that impedance properties *mediated* by HCN channel could be significantly *modulated* by the presence of other sub-threshold conductances and by morphological characteristics (Hutcheon and Yarom, 2000; Hu et al., 2002, 2009; Narayanan and Johnston, 2008; Zemankovics et al., 2010; Rathour and Narayanan, 2012a; Dhupia et al., 2015; Rathour et al., 2016). While these studies elucidated the role of individual conductances in modulating sub-threshold impedance properties, computational frameworks have provided direct lines of evidence for degeneracy (Figures 2B,C) in active dendritic spectral tuning (Rathour and Narayanan, 2012a, 2014).

Similar to sub-threshold spectral tuning properties, although there are specific channels that *mediate* suprathreshold frequency selectivity, it has been shown that the presence of other conductances could critically *modulate* suprathreshold spectral tuning (Reyes et al., 1994; Richardson et al., 2003; Badel et al., 2008; Kispersky et al., 2012; Stark et al., 2013; Das and Narayanan, 2015). Although systematic searches involving multiple channel and morphological properties have not been performed (similar to analyses with subthreshold resonance), these observations strongly postulate degeneracy in suprathreshold spectral tuning, whereby similar selectivity profiles could be achieved through disparate parametric combinations.

## Implications for Active Dendritic Spectral Tuning

Evidence for the role of plastic active dendrites in neuronal computations and cognitive function has been accumulating from multiple explorative efforts employing robust experimental and theoretical tools. The demonstration of spectral tuning in neuronal dendrites allows us to ruminate over functions it would impart to neurons in addition to serving as a frequency-specific *reader* of upstream network activity (Narayanan and Johnston, 2007; Buzsáki, 2010). Presence of distinct sub- and supra-threshold tuning properties suggests that the neuron might be serving as a *correspondent* of temporally parsed activity. The ability to perform spectral selectivity at both regimes with common mechanisms mediating them imparts a capacity to simultaneously *decode* and *encode* information (Bialek et al., 1991) in a network using a robust, degenerate and plastic cellular machinery. Location-dependence of spectral selectivity would endow the neuron with the ability to process spatially segregated inputs carrying salient information in the form of temporally distinct parcels of activity (Colgin et al., 2009), differentially employing multiple operational modes of a single neuron (Poirazi et al., 2003; Narayanan and Johnston, 2012; Ratté et al., 2013; Das and Narayanan, 2015). In addition, the presence of gradients in resonating conductances results in location-dependent phase leads in theta frequency local field potentials (Sinha and Narayanan, 2015), an enhancement in the associated phase coherence (Sinha and Narayanan, 2015) through constriction of the coincidence detection window (Das and Narayanan, 2015), frequency selectivity in local field potentials (Ness et al., 2016) and a location-independent somatic synchronization of input oscillations (Vaidya and Johnston, 2013). Together, the consortium of cellular mechanisms mediating plasticity in expression profiles of these ion channels could result in dynamic tuning of single neurons to network activity of different frequencies in accordance with the behavioral and motivational state of the animal and allow a seamless adaptation to changing salient environmental stimuli. This would have significant ramifications for the function of a single neuron and its dendrites through either a local remapping of spectral tuning property dictating the neuron’s response to a specific input or a global remapping altering the membership of a neuron in an assembly (Buzsáki, 2010; Sinha and Narayanan, 2015).

## Future Directions

Investigations into the field of active dendritic spectral tuning have barely revealed the tip of the iceberg. Experimental studies have clearly demonstrated the role of some VGICs in mediating subthreshold resonance, but more are yet to be unearthed with a clear need to demonstrate the complex spatiotemporal interactions between VGICs (Hu et al., 2002; Rathour and Narayanan, 2012a,b; Das and Narayanan, 2015; Rathour et al., 2016), including their interactions with metabotropic channels and receptors. Suprathreshold spectral tuning has been less conclusively explored, with various efforts marred by the lack of a single well-defined metric to study it. Computational studies have demonstrated that the STA and STA-derived metrics could be used to perform similar quantifications as impedance analysis (Ratté et al., 2013; Das and Narayanan, 2014, 2015). So an important future direction would be an application of these metrics to experimental data comprising somatic and dendritic recordings and ascertain the VGIC- and location-dependence of suprathreshold spectral tuning in neurons. A second avenue of investigation would be plasticity in spectral tuning which has been demonstrated for subthreshold resonance (Narayanan and Johnston, 2007, 2008) and it would be critical to demonstrate plasticity in suprathreshold tuning. In this regard, the role of various metabotropic receptors and signaling molecules and their cogent interactions present an important question which could be dissected using a combination of computational models and experiments employing various well-established plasticity protocols. A third line of investigation would be neuromodulation. Variability in circuits and function in response to changing behavioral states of animals has largely been attributed to neuromodulatory networks (Hasselmo, 1995; Marder et al., 2014). Together, these distinct directions along with established changes in spectral selectivity, neuromodulation and oscillations under physiological and pathophysiological conditions (Buzsáki, 2006; Brager and Johnston, 2007, 2014; Narayanan and Johnston, 2007, 2008; Shin et al., 2008; Marcelin et al., 2009; Narayanan et al., 2010; Traub and Whittington, 2010; Wang, 2010; Brager et al., 2012; Do et al., 2012; Marder et al., 2014; Zhang et al., 2014) lead to the pivotal question of how single neuron spectral selectivity, its location-dependence and plasticity contribute to information processing under *in vivo* conditions.

## Author Contributions

AD, RKR and RN drafted the manuscript, revised it critically for important intellectual content and approved the final version of the manuscript.

## Funding

The work reviewed here was supported by the Human Frontier Science Program (HFSP) Organization (RN), the Department of Biotechnology (RN), the Department of Science and Technology (RN), a Bristol Myers Squibb fellowship (AD) and the Indian Institute of Science (AD, RKR and RN).

## Conflict of Interest Statement

The authors declare that the research was conducted in the absence of any commercial or financial relationships that could be construed as a potential conflict of interest.
